# EXTENDING FINDINGS FROM AGING COHORTS TO BROADER POPULATIONS OF INTEREST: CONSIDERING TRANSPORTABILITY

**DOI:** 10.1093/geroni/igae098.0532

**Published:** 2024-12-31

**Authors:** Eleanor Hayes-Larson

**Affiliations:** USC Leonard Davis School of Gerontology, Los Angeles, California, United States

## Abstract

Aging cohorts and other commonly-used data sources are typically not the populations of interest for scientific inferences in epidemiologic aging research. Rather, inferences are desired about particular target populations (e.g. defined by geography, age, birth cohort, or medical history), making external validity of study findings a particularly relevant concern. Recent developments in epidemiologic methods facilitate quantitative assessment of external validity and offer statistical solutions when study samples are not representative of the target population of interest. In this presentation, we discuss approaches to evaluating external validity in aging research, and methods for extending findings to a given target population when external validity appears threatened. We emphasize estimators that are valid for lifecourse research questions, and offer several recent examples from cognitive aging research.

